# Upregulation of miR-382 contributes to renal fibrosis secondary to aristolochic acid-induced kidney injury via PTEN signaling pathway

**DOI:** 10.1038/s41419-020-02876-1

**Published:** 2020-08-14

**Authors:** Xiaoyan Wang, Ning Xue, Shuan Zhao, Yiqin Shi, Xiaoqiang Ding, Yi Fang

**Affiliations:** 1 Department of Nephrology, Zhongshan Hospital, Fudan University, Shanghai, China; 2 Shanghai Medical Center of Kidney, Shanghai, China; 3 Shanghai Institute of Kidney and Dialysis, Shanghai, China; 4 Key laboratory of Kidney and Blood Purification, Shanghai, China

**Keywords:** Necroptosis, Cell death and immune response, Transforming growth factor beta

## Abstract

Acute kidney injury (AKI) has a critical role in the development of chronic kidney disease (CKD). Building on our previous findings, we explored the role of miR-382 in facilitating the transition of AKI to CKD using the Aristolochic acid (AA) nephropathy model, which was induced by intraperitoneal injection of aristolochic acid I salt (10 or 20 mg/kg). The effects of genetic depletion, pharmacologic inhibition, or overexpression of miR-382 on the PTEN/AKT signaling pathway were examined in vivo and in vitro. Changes in renal pathology and renal epithelial polarity were evaluated. A luciferase reporter assay was performed to investigate the reciprocal suppression relationship between miR-382 and *PTEN*. Renal fibrosis developed 14 d after AA exposure in a dose- and time-dependent manner. Renal abundance of miR-382 was upregulated following AA treatment, while genetic depletion or pharmacological inhibition of miR-382 partially reversed renal tubulointerstitial fibrosis. Expression of PTEN, a target of miR-382, was downregulated and subsequently its downstream AKT signaling pathway was activated during AKI to CKD transition induced by AA. Inhibition of PTEN in vitro resulted in the acquisition of the EMT phenotypes. Furthermore, upregulation of miR-382 in renal epithelial cells was partially mediated by the activation of NF-kB signaling, with a substantial elevation of proinflammatory cytokines. An in vivo study revealed that either miR-382 knockdown or miR-382 knockout was pivotal for inflammatory suppression, while an in vitro experiment confirmed that upregulation of miR-382 in cultured MTEC cells under AA exposure was remarkably reversed by NF-kB siRNA. These data indicated a novel role for the NF-κB/miR-382/PTEN/AKT axis in the pathogenesis of tubulointerstitial fibrosis following AA-induced acute renal tubular epithelial injury. Targeting miR-382 may lead to a potential novel therapeutic approach for retarding the AKT to CKD transition.

## Introduction

Acute kidney injury (AKI), a syndrome defined by rapidly declining renal function, has a high worldwide incidence. Although AKI was considered a reversible disease, recent clinical research has revealed that even patients who have completely recovered from an episode of AKI are at a higher risk for chronic kidney disease (CKD)^1,2^. The proximal tubular cells are reported as the primary target as well as a dominant trigger of injury and progression of kidney disease^3,4^. Tubular epithelial cells are the determinant in the development of interstitial inflammation^5^, which can be observed in the early stage of many renal diseases^6^. However, mechanisms underlying the transition from AKI to CKD remain largely unclear. Aristolochic acid nephropathy (AAN), a common model of AKI to CKD transition secondary to renal toxicity^7,8^, is an important drug-associated renal injury disease^9^ and AA mainly targets tubular epithelial cells and promotes death of tubules via formation of DNA adducts^9,10^.

MicroRNAs are endogenous, small non-coding RNAs, ~22 nucleotides in length, which mostly regulate gene expression negatively at the post-transcriptional level and have a critical role in kidney diseases^11,12^. MiR-382, located at chromosome 14q32.31, is a member of the miR-17-92a cluster. Microarray analyses have revealed that miR-382 is upregulated during TGF-β1-induced epithelial to mesenchymal transition (EMT) in human renal epithelial cells^13^ and also contributed to the development of renal fibrosis in mouse unilateral ureteral obstruction (UUO) models^14,15^. Thus, although it appeared that miR-382 has a critical role in CKD progression, whether miR-382 contributes to AKI-induced CKD remained unclear.

Phosphatase and tensin homologs deleted on chromosome 10 (PTEN)/protein kinase B(AKT) pathway has been reported in tumors as a tumor suppressor^16,17^. In addition, depletion of PTEN was characteristic of renal fibrosis and overexpression of PTEN expression attenuated tubulointerstitial fibrosis^18–22^, inhibited macrophage polarization from M1 to M2^23,24^ and suppressed inflammation responses^25,26^. Given that PTEN has been identified as a target of miR-382 in liver generation, acute promyelocytic leukemia, and tumor angiogenesis as well as oxidative stress of the tubular epithelium^27–30^, which remains unclear in kidney fibrosis. NF-κB is an important transcription factor that mediates the expression of various miRNAs^31–34^ and is also a pivotal mediator of inflammation^35^. Therefore, we hypothesized that activation of NF-kB may induce transcription of miR-382, which promotes the AKI to CKD transition by targeting PTEN/AKT signaling.

## Results

### AA induces miR-382 overexpression and AKI-CKD transition in mice

**Fig. 1:**
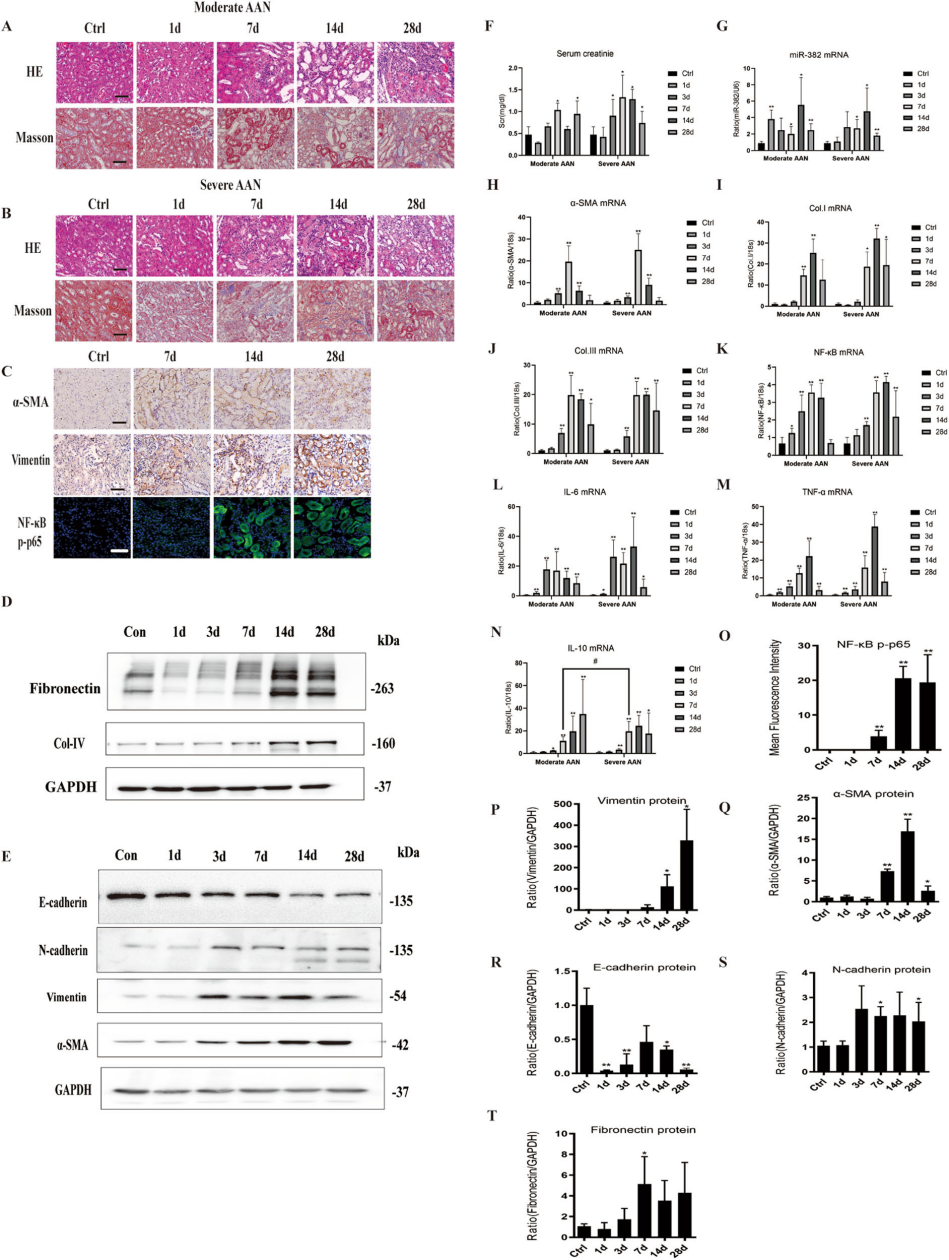
AA-induced miR-382 expression and development of AKI to CKD in mice. **a** Representative images of mice kidney sections from Moderate AAN group administered 10 mg/kg of AA with H&E and Masson staining. Scale bars, 100 μm. **b** Representative images of mice kidney sections from the Severe AAN group administered 20 mg/kg AA with H&E, Masson staining. Scale bars, 100 μm. **c** Immunostaining for α-SMA and Vimentin from control mice, days 7, 14, and 28 in the severe AAN group. Scale bars, 100 μm. Immunofluorescence for p-NF-κB p65 from control mice, days 7, 14, and 28 in the severe AAN group. Scale bars, 50 μm. **d**, **e** Representative immunoblot analysis of Fibronectin, Collagen IV, E-cadherin, N-cadherin, Vimentin, and α-SMA in kidney tissues from the severe AAN group. GAPDH served as the standard. **f** Renal dysfunction of AAN was determined in AA-induced AKI-to-CKD models. Serum creatinine (Scr) was measured in sera; *n* = 6 per group. **g** Relative abundance of miR-382 was examined in mice kidney tissues. **h**–**j** Renal fibrotic manifestation was examined in the models. Relative mRNA levels of α-SMA, Collagen I and Collagen III in mice kidney tissues were examined; *n* = 6 per group. **k** Relative mRNA levels of NF-κB in mice kidney sections from AA-induced nephropathy; *n* = 6 per group. **l**–**n** Renal inflammation manifestation was examined in the models. Relative mRNA levels of IL-6, IL-10, and TNF-α in mice kidney tissues were examined; *n* = 6 per group. **o** Quantification of mean Fluorescence intensity for p-NF-κB p65 from control mice, day 1, 7, 14, and 28 in severe AAN group. We randomly choose five microscopical field per sections, *n* = 3 mice per group. **p**–**t** Quantification of western blot analysis for Vimentin, α-SMA, E-cadherin, N-cadherin, and Fibronectin from control mice, days 1, 3, 7, 14, and 28 in the severe AAN group. *P < 0.05; **P < 0.01; ANOVA.

In order to observe the natural course of AKI to CKD transition, a time- and dose- course study was performed in mouse models of Aristolochic acid Nephropathy (1, 3, 7, 14, and 28 days; moderate versus severe). AA related kidney injury was characterized by a significant loss of proximal tubule brush, severe atrophy of the proximal tubular epithelium (PTE), acute tubular necrosis and obvious extracellular matrix (ECM) deposition in the mesenchyme. In comparison with the SAAN group, more proliferative PTE cells were seen in the MAAN group (Fig. 1a, b). EMT-related mesenchymal biomarkers Vimentin, α-smooth muscle actin (α-SMA) and N-cadherin^36^ were increased while epithelial biomarkers E-cadherin was decreased on days 7, 14, and 28, as detected by immuno-histological staining and western blot. (Fig. 1c, e, p–s) Fibrotic related proteins fibronectin and collagen IV were increased in SAAN group on days 14 and 28 (Fig. 1d, t). Serum creatinine (Scr) ascended significantly on days 7 (MAAN group) and 3 (SAAN group), compared with that in the control group (Fig. 1f). Renal abundance of miR-382 was elevated early on day 1 (MAAN group), while it did not reach statistical significance until day 7 in the SAAN group, indicating that abundance of miR-382 might not be associated with the dosage of AA (Fig. 1g). Concomitantly, mRNA expression of NF-κB was elevated since day 1 and peaked on day 7 (MAAN group), and on day 14 in the SAAN group (Fig. 1k). Activity of NF-κB phosphorylated p65 in renal section was enhanced significantly on days 14 and 28 in the SAAN group (Fig. 1c, o). A similar tendency was found in the mRNA expression of fibrotic genes, such as *α-SMA, Collagen I*, and *Collagen III*, as well as inflammatory cytokines including *interleukin-6* (*IL-6*), *interleukin-10* (*IL-10*), and *tumor necrosis factor-α* (*TNF-α*); (Fig. 1i–n). These results demonstrated that activation of NF-κB and upregulation of miR-382 was accompanied by the aggravation of inflammatory infiltration, progression of EMT and development of renal fibrosis.

### Knockout of miR-382 reverses renal inflammation and fibrosis in mice

**Fig. 2:**
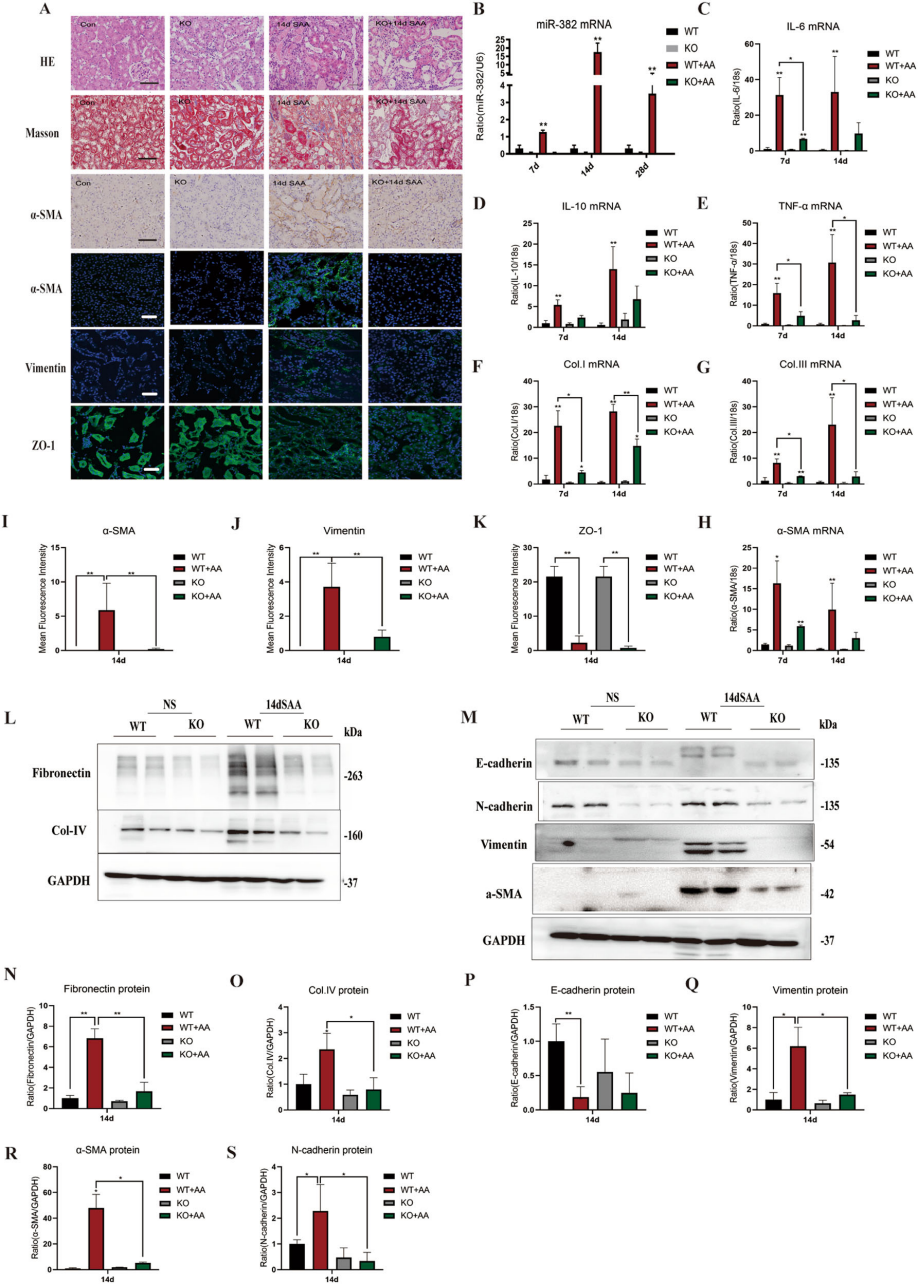
Knockout of miR-382 contributes to reverse renal inflammation and fibrosis in mice. **a** Representative images of kidney sections from miR-382 WT or KO mice 14 d after the administering of 20 mg/kg AA (SAA group) with H&E and Masson staining, immunostaining for α-SMA as well as immunofluorescence for α-SMA, Vimentin and ZO-1. Scale bars, 100 or 50 μm. **b** Quantification of relative mRNA levels of miR-382 in mice kidney from miR-382 WT or KO mice 7, 14, and 28 days after the administering of 20 mg/kg AA; *n* = 6 per group. **c**–**e** Relative mRNA levels of IL-6, IL-10, and TNF-α in mice kidney tissues were examined in these groups. *n* = 6 per group. **f**–**h** Inhibition of miR-382 in mice reversed fibrotic manifestation of kidney significantly. Relative mRNA expression of Collagen I, Collagen III, and α-SMA in mice kidney tissues were examined between miR-382 WT and KO group 7 and 14 d following the administering of 20 mg/kg AA. *n* = 6 in each group. **i**–**k** Quantification of mean Fluorescence intensity for α-SMA, Vimentin, and ZO-1 between miR-382 WT and KO group 14 d following the administering of 20 mg/kg AA. We randomly choose five microscopical field per sections, *n* = 3 mice per group. **l**, **n**, **o** Representative and quantification immunoblot analysis of Fibronectin and Collagen IV in renal tissue from miR-382 WT or KO mice 14 d following 20 mg/kg AA treatment. GAPDH served as the standard; *n* = 6 in each group. **m**, **p**–**s** Representative and quantification immunoblot analysis of E-cadherin, N-cadherin Vimentin, and α-SMA in renal tissue from miR-382 WT or KO mice 14 d following 20 mg/kg AA treatment. GAPDH served as the standard; *n* = 6 in each group. *P < 0.05; **P < 0.01; ANOVA.

Relevance of miR-382 upregulation and development of renal fibrosis was further investigated in miR-382 knockout mice. A dose of 20 mg/kg AA was intraperitoneally administered to miR-382 knockout (KO) mice as well as wildtype (WT) mice (*n* = 3 per group) and mouse were killed at day 14. Firstly, miR-382 KO mice showed significantly less renal lesions, a mild loss of proximal tubule brush, less proximal tubule atrophy and less extracellular matrix (ECM) deposition than WT mice (Fig. 2a). Compared with WT mice, expression of α-SMA and Vimentin were decreased and loss of epithelial biomarker ZO-1 was attenuated in miR-382 KO mice after AA treatment (Fig. 2a, i–k). MiR-382 upregulation was significantly abolished in miR-382 KO mice compared to WT mice (Fig. 2b). Moreover, increased mRNA expression of α-SMA, Collagen I and Collagen III, and protein expression of fibronectin, collagen IV, N-cadherin, α-SMA and vimentin as well as loss of E-cadherin secondary to AA stimulation was found to be markedly attenuated by miR-382 KO (Fig. 2f–h, l–q). In addition, compared to the anti-scramble group, anti-miR-382 Oligos treatment were also proved to be reno-protective (Supplementary Fig. S1).

### MiR-382 promotes renal fibrosis via PTEN-mediated AKT signaling

**Fig. 3:**
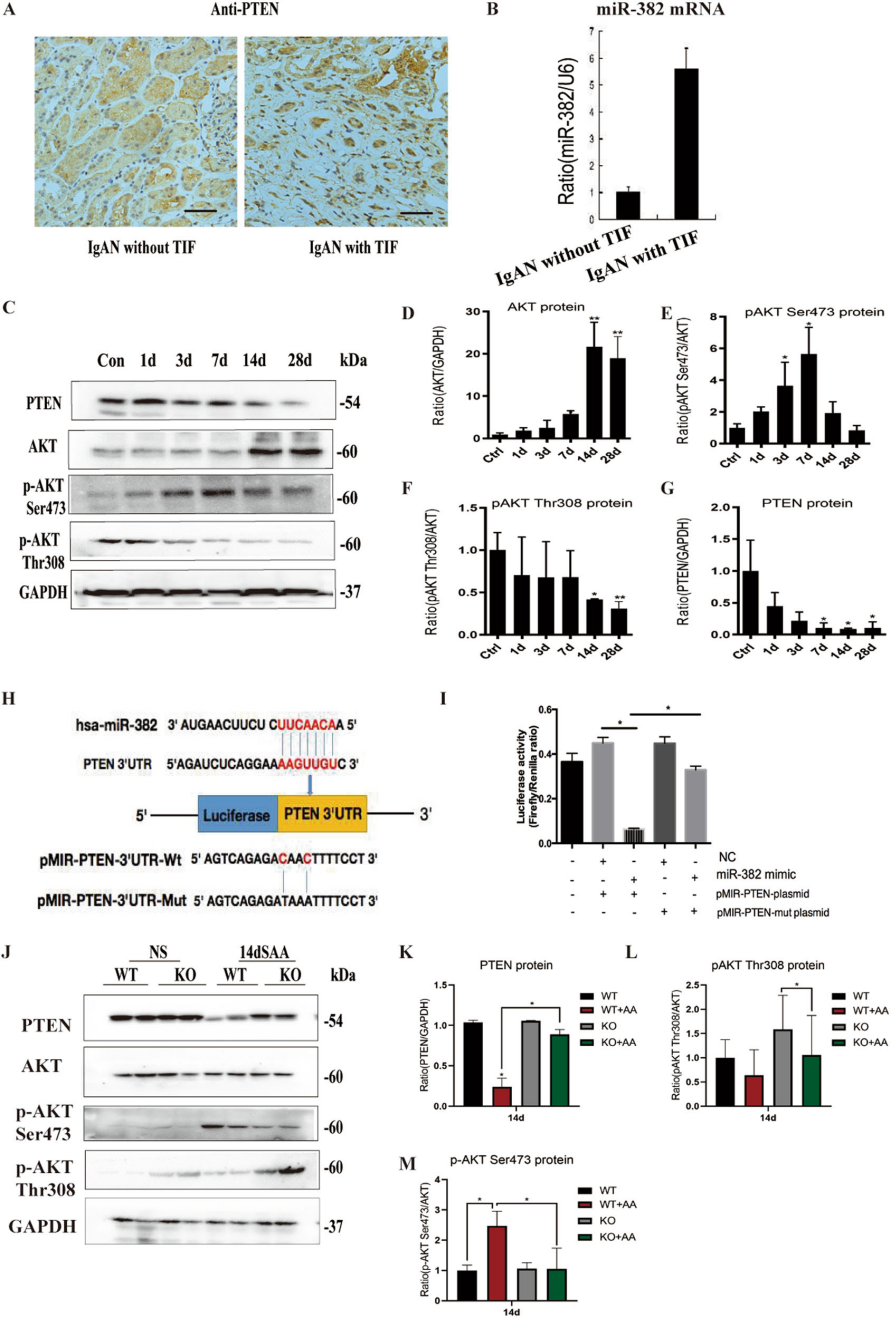
MiR-382 promotes renal fibrosis via PTEN-mediated AKT signaling in AA reduced AKI to CKD. **a** Representative image of immunohistochemical staining for PTEN of human renal biopsy specimens from patients with IgAN with or without tubulointerstitial fibrosis. **b** Relative expression of miR-382 in human renal biopsy tissues. *n* = 6 per group. **c**–**g** miR-382 suppressed PTEN and activated AKT signaling pathway. Representative and quantification immunoblot analysis of PTEN, AKT, p-AKT Ser473, and p-AKT Thr308 in time course study of SAAN. GAPDH served as standard of PTEN; AKT served as standard of p-AKT; n-6 per group. **h** The targetScan bioinformatics website was used to predict the possible sequence of PTEN 3′UTR combined with miR-382. Mutation was induced in the PTEN 3′UTR sequence. **i** luciferase activity was quantified in 293 T cells between the Control group, NC+pMIR-PTEN-plasmid group, miR-382 mimic+ pMIR-PTEN-plasmid group, NC+pMIR− PTEN-mut plasmid group and miR-382 mimic+pMIR-PTEN− mut plasmid group. Dual-luciferase activity was measured via a Dual-Glo Luciferase Assay System. *n* = 3 per group. **j**–**m** Representative and quantification immunoblot analysis of PTEN, AKT, p-AKT Ser473, and p-AKT Thr308 in mice renal from miR-382 WT or KO mice 14 d following severe administering of AA. GAPDH served as standard of PTEN; AKT served as standard of p-AKT; n-6 per group. *P < 0.05; **P < 0.01; ANOVA.

Given that transition of AKI to CKD was induced by the upregulation of miR-382 in the renal tissue of mouse AA models, we explored the mechanisms underlying miR-382 mediated renal fibrosis. Reciprocal suppression between miR-382 and PTEN was verified both in human and mouse kidneys. Notably, depletion of PTEN expression and upregulation of miR-382 were found in IgAN patients with tubulointersitial fibrosis (TIF), compared with IgAN patients without TIF (Fig. 3a, b). In mouse model of AA, PTEN expression was inhibited and its downstream AKT was activated through phosphorylation of AKT on Ser473. However, phosphorylation of AKT on Thr308 was significantly downregulated (Fig. 3c–g). A dual-luciferase reporter assay was performed (Fig. 3h). Luciferase activity of the 293T cells was significantly suppressed when co-transfecting the miR-382 mimics and pMIR-PTEN-plasmid, but not with the pMIR-PTEN-mut plasmid (mutation has occurred in the selected sequence), suggesting that miR-382 inhibits PTEN by combining with 3′UTR of *pten* (Fig. 3i). Furthermore, inhibition of PTEN and activation of AKT through Phosphate-AKT Ser 437 were attenuated in miR-382 KO mice, which provided robust evidence of reciprocal suppression between miR-382 and PTEN (Fig. 3j–m). Consider together, PTEN/AKT signaling might get involved in the contribution of miR-382 in driving AKI-to-CKD after AA treatment.

### AA induces miR-382 expression and promotes EMT in cultured mouse tubular epithelial cells

**Fig. 4:**
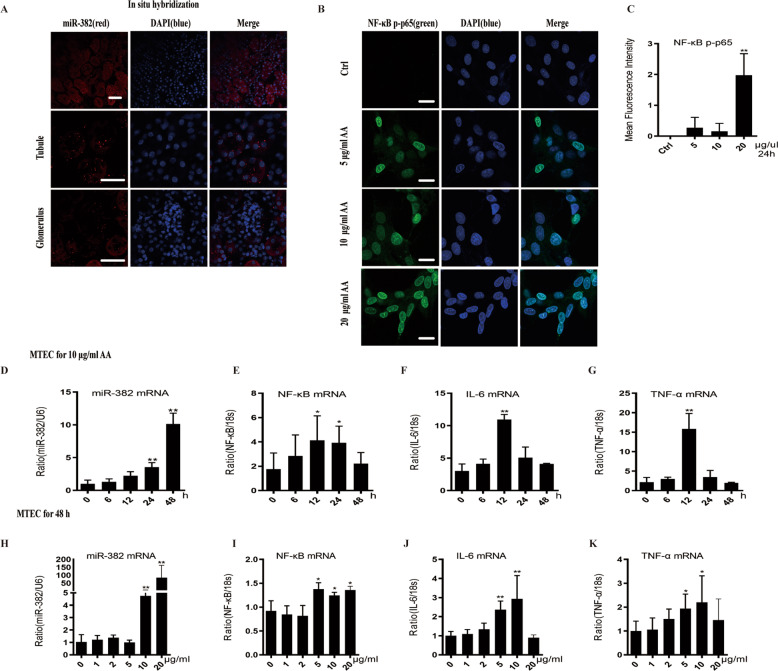
Distribution of miR-382 in mice kidney and AA-induced inflammation in mTECs. **a** In situ hybridization of miR-382 in mice kidney samples was performed. Representative images of miR-382 ISH in tubule and glomerulus. miR-382 was visualized by staining with Dye3 (Red). Nuclei were counterstained with DAPI (blue). Scale bars, 100 μm. **b**, **c** Immunofluorescence images and quantification of mean Fluorescence intensity for p-NF-κB p65 from control cells or after treatment with 5, 10, and 20 μg/ml AA for 24 h in mTECs. We randomly choose five microscopical field per sections, *n* = 3 holes per group. Scale bars, 50 μm. **d** Relative abundance of miR-382 in mTECs following treatment with 10 μg/ml AA for 0, 6, 12, 24, and 48 h. U6 served as the standard. *n* = 6 per group. **e**–**g** Quantification of relative mRNA levels of IL-6, TNF-α, and NF-κB in vitro in these groups; *n* = 6 per group. **h** Relative abundance of miR-382 in mTECs following treatment with 0, 1, 2, 5, 10, and 20 μg/ml of AA for 48 h. U6 served as the standard; *n* = 6 per group. **i**–**k** in vitro Quantification of relative mRNA levels of IL-6, TNF-α and NF-κB in these groups. *n* = 6 per group. *P < 0.05; **P < 0.01; ANOVA.

To confirm miR-382 localization in the kidney, we performed in situ hybridization (ISH) of miR-382 in mouse kidney sections. miR-382 is widely distributed in the cortex and the medulla of the kidney, especially in the PTE while expression in the glomeruli were relatively sparse (Fig. 4a). As mentioned above, renal tubules are dominant in response to injuries^5^. Therefore, we performed a time course study and a dose-response study in mouse renal tubular epithelial cells (mTECs). Activity of NF-kB phosphorylated p65 in mTECs was increased in a dose-dependent way after AA stimulation (Fig. 4b, c). The abundance of miR-382 increased either in a time-dependent manner (Fig. 4d) or in a dose-dependent manner (Fig. 4h). A significant increase in mRNA expression of IL-6, TNF-α, and NF-κB were detected after treating with AA ranging from 5 to 20 μg/ml for 48 h (Fig. 4e–g) or with AA (10 μg/ml) for 12 h (Fig. 4i–k). Above all, AA stimulation in mTECs resulted in activation of NF-κB, upregulation of miR-382, and increased expression of inflammatory cytokines.

**Fig. 5:**
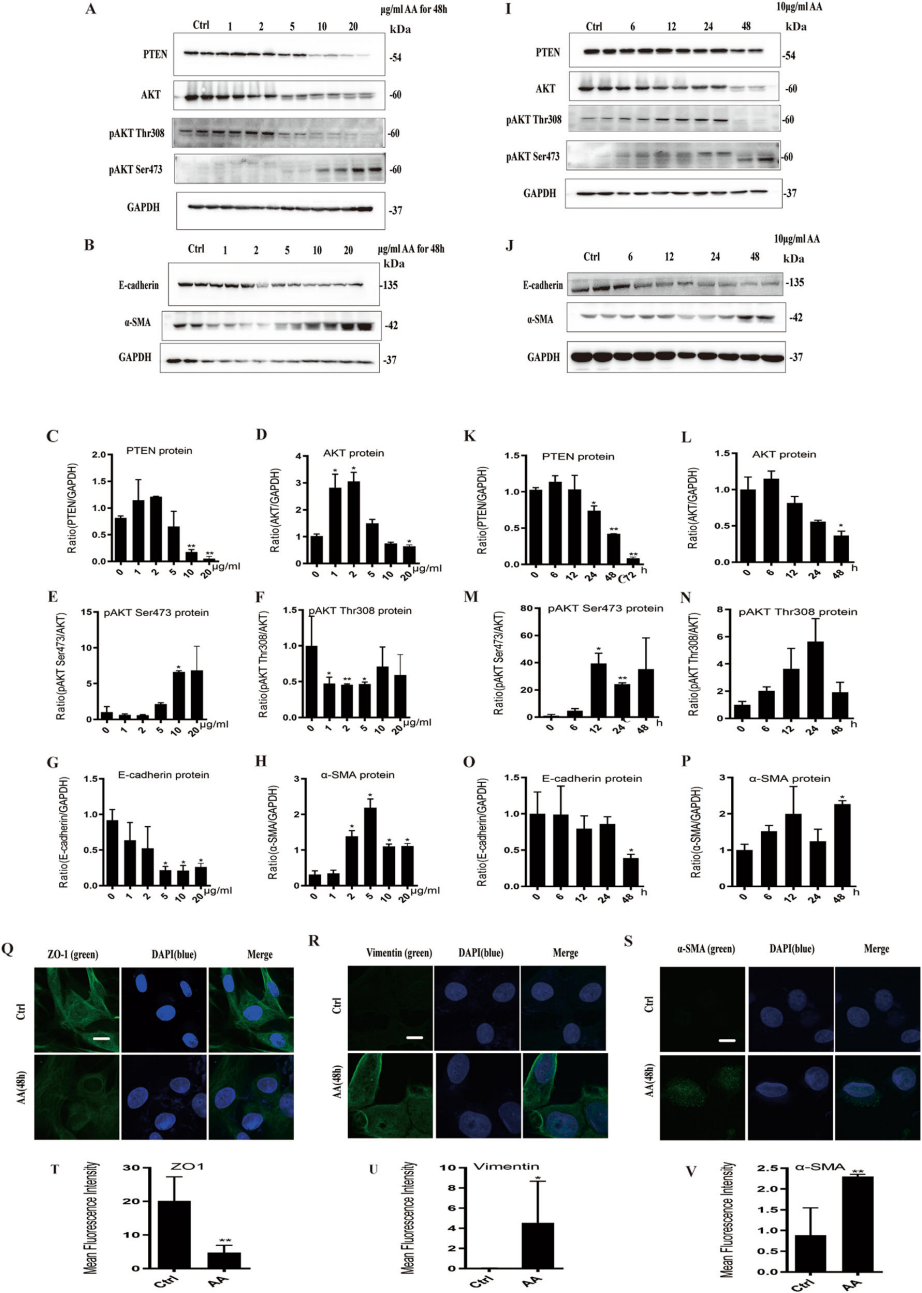
AA stimulation regulated PTEN/AKT signaling pathway and promoted EMT in mTECs. **a**, **c**–**f** Representative and quantification immunoblot analysis of PTEN, AKT, p-AKT Thr308, and p-AKT Ser473 in mTECs from administration of 0, 1, 2, 5, 10, and 20 μg/ml AA for 48 h. GAPDH served as the standard of PTEN, p-AKT served as the standard of AKT; *n* = 6 per group. **b**, **g**–**h** Representative and quantification immunoblot analysis of E-cadherin, N-cadherin, and in mTECs from administration of 0, 1, 2, 5, 10, and 20 μg/ml AA for 48 h. GAPDH served as the standard of PTEN, p-AKT served as the standard of AKT; *n* = 6 per group. **i**, **k**–**n** Representative and quantification immunoblot analysis of PTEN, AKT, p-AKT Thr308, and p-AKT Ser473 in mTECs following the administering of 10 μg/ml AA for 0, 6, 12, 24, and 48 h separately. GAPDH served as the standard of PTEN, p-AKT served as the standard of AKT; *n* = 6 per group. **j**, **o**–**p** Representative and quantification immunoblot analysis of E-cadherin, N-cadherin, and in mTECs following the administering of 10 μg/ml AA for 0, 6, 12, 24, and 48 h separately. GAPDH served as the standard; *n* = 6 per group. **q**–**v** Immunofluorescence images and quantification of mean fluorescence intensity for ZO-1, Vimentin, and α-SMA from control group and after treatment with 10 μg/ml AA for 48 h in mTECs. We randomly choose five microscopical field per sections, *n* = 3 holes per group. Scale bars, 10 μm. *P < 0.05; **P < 0.01; ANOVA.

PTEN/AKT signaling and phenotype of EMT were examined in AA-induced tubular epithelial cell injury. In consistent with in vivo study, protein expression of PTEN was significantly inhibited when mTECs were incubated with AA (10 and 20 μg/ml) either for 24 or for 48 h. Phosphorylation of AKT on Ser473 was increased in a dose- and time-dependent way while phosphorylation of AKT on Thr308 was decreased (Fig. 5a, j, c–f, l–o). Furthermore, E-cadherin was downregulated while α-SMA was upregulated after treatment of AA with indicated concentration for 48 h (Fig. 5b, k, g, h, p, q). As detected by immunofluorescence staining, expression of epithelial biomarker ZO-1 was declined and mesenchymal biomarker Vimentin and α-SMA were upregulated after AA (10 μg/ml) treatment for 48 h (Fig. 5r–w). Overall, AA activated inflammation and subsequently upregulated miR-382, thereby promoting PTEN/AKT signaling and EMT in a time- and dose-dependent manner. This was consistent with the results of the in vivo study.

### AA induces miR-382 overexpression via NF-κB activation and promotes EMT mediated by PTEN signaling

**Fig. 6:**
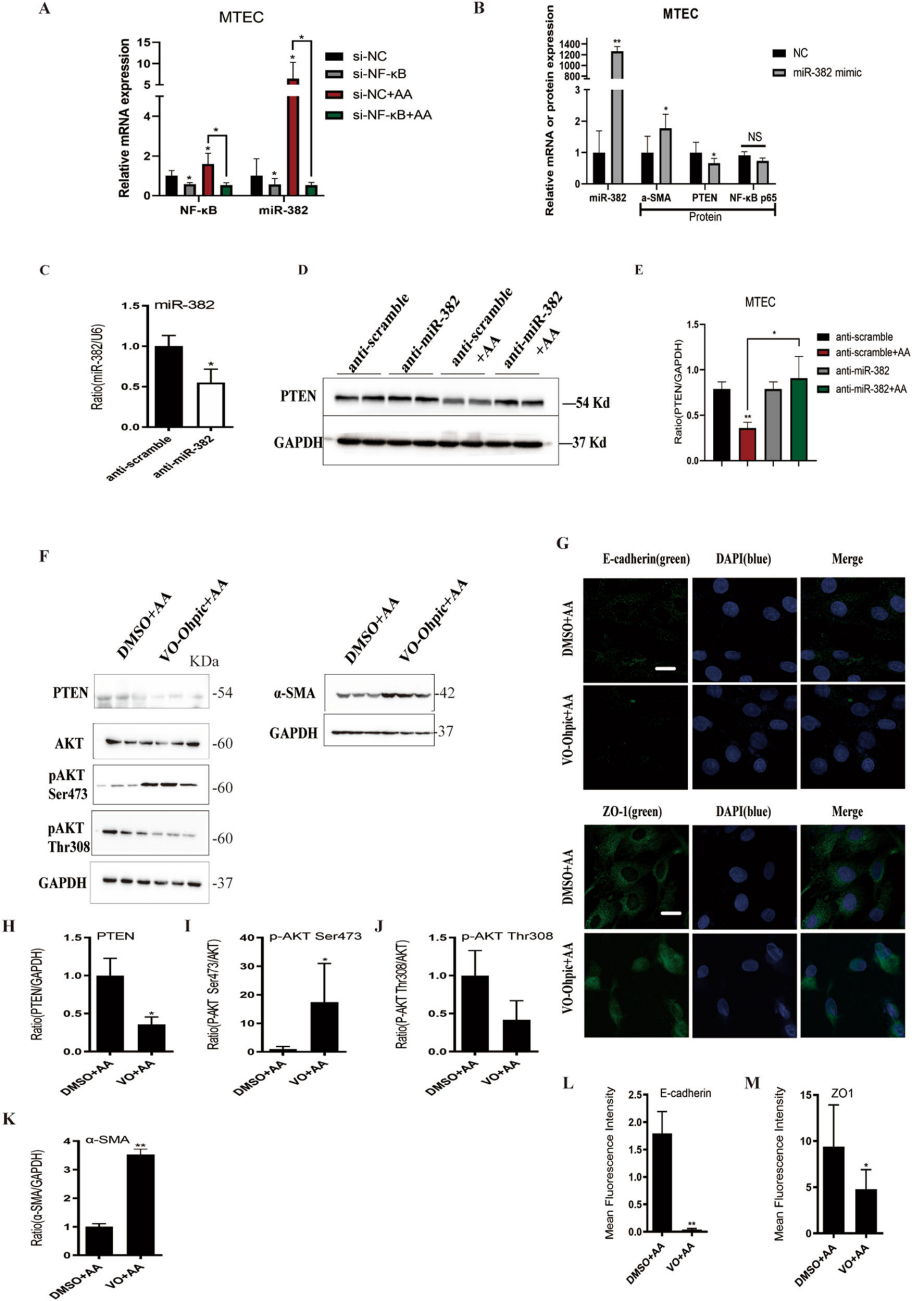
Overexpression or suppression of miR-382 and inhibition of NF-κB or PTEN in mTECs. **a** Relative mRNA levels of NF-κB and miR-382 in si-NC, si-NF-κB, si-NC + AA (10 μg/ml AA; 48 h), and si-NF-κB + AA groups. U6 and 18s served as standard separately; *n* = 6 per group. **b** Relative mRNA of miR-382 and protein of α-SMA, PTEN, and p65 in miR-382 mimic (100 nM) or negative control (NC) transfection in mTECs. U6 and GAPDH served as standard separately; *n* = 6 per group. **c** The effect of anti-miR-382 in mTECs was confirmed by qPCR of miR-382 between anti-scramble group and anti-miR-382 group. **d**, **e** Representative and quantification immunoblot analysis of PTEN in anti-scramble, anti-miR-382 (100 nM), anti-scramble + AA (10 μg/ml AA, 48 h) and anti-miR-382 + AA groups. GAPDH served as standard separately; *n* = 6 per group. **f** The impact of PTEN on EMT was confirmed by comparison with DMSO + AA (10 μg/ml AA, 48 h) and VO-Ohpic(PTEN inhibitor)+ AA (10 μg/ml AA, 48 h) groups. Representative immunoblot analysis of PTEN, AKT, p-AKT Ser473, p-AKT Thr308, and α-SMA in DMSO + AA (10 μg/ml AA, 48 hours) and VO-Ohpic (PTEN inhibitor)+ AA (10 μg/ml AA, 48 h) groups. GAPDH served as standard separately; *n* = 6 per group. **g** Immunofluorescence images for E-cadherin and ZO-1, Vimentin and α-SMA from control group and after treatment with 10 μg/ml AA for 48 h in mTECs. We randomly choose five microscopical field per sections, *n* = 3 holes per group. Scale bars, 10 μm. **h**–**m** Quantification of western blot analysis of PTEN, p-AKT Ser473, p-AKT Thr308, α-SMA, and N-cadherin and mean fluorescence intensity of E-cadherin and ZO-1 between DMSO + AA group and VO + AA group. *P < 0.05; **P < 0.01; ANOVA.

According to previous studies, NF-κB is essential to mediate inflammation response and also acts as a transcription factor of a variety of miRNAs. NF-κB siRNA treatment significantly attenuated AA-induced activation of NF-κB, and markedly downregulated miR-382, which provided evidence that NF-κB might regulate miR-382 (Fig. 6a). Furthermore, overexpression of miR-382 inhibited PTEN abundance and upregulated α-SMA expression, it did not exert any effects on NF-κB p65 expression, which indicated NF-κB did not respond to miR-382 overexpression (Fig. 6b). In addition, Expression of PTEN, which was consistently reduced following treatment with AA (10 μg/ml for 48 h), was reversed following anti-miR-382 treatment, which verified reciprocal suppression between miR-382 and PTEN in vitro (Fig. 6c–e). Moreover, PTEN was inhibited with PTEN inhibitor OV-Ohpic trihydrate prior to AA treatment. Inhibition of PTEN followed by AA treatment, the effects on inhibition of PTEN, phosphorylation of AKT on Ser473 and downregulation of p-AKT Thr308 were enhanced, compared with DMSO + AA group. Besides, downregulation of E-cadherin and ZO-1 and upregulation of α-SMA were aggravated in the OV-Ohpic + AA group, which indicated that PTEN/AKT signaling pathway is involved in the development of EMT in renal tubular epithelial cells (Fig. 6f–g, h–m).

## Discussion

This study provided novel evidence showing that the non-coding small RNA, miR-382, mediates TIF in mouse AA-induced AKI-to-CKD models. We have shown that miR-382 acted as a critical regulator of the inflammation response and progression of EMT and accumulation of ECM, both in vivo and in vitro. Based on the results of a dual-luciferase assay, we proposed a molecular mechanism whereby PTEN, an important factor that inhibits renal fibrosis, serves as a downstream target of miR-382. Furthermore, our findings indicated that AKI-to-CKD transition induced by AA was partially mediated by the activation of miR-382/PTEN/AKT signaling. In vivo and in vitro inhibition of miR-382, via genetic or pharmacological methods, rescued the loss of PTEN, negatively regulated the immune response, reversed EMT, and attenuated the accumulation of ECM. In contrast, overexpression of miR-382 resulted in the converse effect. Moreover, our result revealed the molecular mechanism by which NF-κB acts as an upstream transcription factor of miR-382 during AA-induced AKI-to-CKD transition.

The close association between Chinese herbs containing aristolochic acids (AA) and rapidly progressive interstitial renal fibrosis in humans has been reported in several countries during the last decade^37^. This murine AAN model was characterized by the development of significant interstitial fibrosis, prominent tubular atrophy, and necrosis, which is identical to AAN seen in humans^38,39^ and shares some features, such as upregulation of miR-382, loss of PTEN expression, and impaired renal function.

As mentioned above, renal miR-382 upregulation was accompanied by the development of interstitial fibrosis as well as degeneration and regeneration of tubular epithelium, which is consistent with previous studies^13–15^. However, circulating miR-382 in mice seems to be significantly decreased in drug-or vascular calcification-induced kidney injury models^28,40^, which opposes the expression of miR-382 in renal tissue. To avoid renal toxicity caused by microRNA inhibitors, our study is the first one to establish miR-382 knockout mice and reveal effect of miR-382 on AA-induced chronic kidney injury. However, miR-382 expression is not specific to kidneys and is also seen in various other tissues such as liver, heart, and brain^15^. We performed ISH of miR-382 in mouse renal sections and found that miR-382 is expressed globally in the cortex and the medulla, especially in the proximal tubular epithelium, which supports our in vitro studies using mouse tubular epithelium cell lines.

In addition, our study demonstrated that the loss of PTEN expression in renal fibrosis, which has since been substantiated by many recent studies^14,18,41,42^. Both in vivo and in vitro, phosphorylation of AKT on Ser473 was increased as along with AA-induced kidney injury, which was consistent with previous studies^43^. In addition, we found impaired AKT activation with difference between Ser473 and Thr308 AKT phosphorylation during AKI-CKD transition, which also contributed to myocardial infarct size in 5/6 nephrectomy rats^43^. Therefore, we speculated that mutual regulation between p-AKT Ser473 and Thr308 might exist and phosphorylation of AKT on Ser473 would have a dominant role in PTEN-loss-induced AKT activation. In our in vitro study, inhibition of PTEN increased p-AKT Ser473 and decreased p-AKT Thr308 but had no effect on total AKT. Therefore, other mechanism might exist in the regulation of total AKT.

The reciprocal relationship between miR-382 and PTEN has been reported in acute promyelocytic leukemia, infantile hemangioma, and liver regeneration^27,29,44^ In our study, we performed a dual-luciferase reporter assay, which confirmed that miR-382 negatively regulated PTEN by combining the 3′UTR of gene *pten* in 293T cells. Further, immunohistochemical staining of PTEN and RT-qPCR of miR-382 in renal biopsy sections from patients with IgA nephropathy (with or without TIF) as well as protein expression of PTEN and its downstream AKT in kidney from mouse AAN model both in WT and miR-382 KO mice also substantiated this relationship. Furthermore, we found inhibition of PTEN in vitro partially accelerated EMT progression, which has been reported in diabetic nephropathy^19^ or cancers^45^. However, intervention of PTEN in vivo to further examine its effect on renal fibrotic outcome is absent in our study.

Considerable evidence indicates that renal inflammation has a central role in the initiation and progression of CKD^46^. AA-induced inflammation in kidneys is a type of sterile inflammation, which is defined as inflammation in the absence of infectious agents or specific immunogens^47^. During this process, leukocytes, fibrogenic cells, and resident kidney immune cells are recruited to the renal interstitium, which lead to increased production of proinflammatory cytokines. Administering of a single dose of AA leads to a gradual increase in proinflammatory renal cytokines until day 28, which may account for the pivotal role had by inflammation in the progression from AKI to CKD. Compared that of with WT mice, the prominent expression of inflammatory cytokines in miR-382 KO mice was attenuated following treatment with 20 mg/kg AA, indicating that miR-382 was a proinflammatory microRNA. However, in our in vitro study, the elevation of inflammatory cytokines started 12 h post AA treatment which was prior to the upregulation of miR-382, showing that the inflammation response possibly promoted miR-382 expression during the early stage of AA-induced kidney injury. Thus, AA-activated inflammatory reaction led to the upregulation of miR-382, a proinflammatory microRNA, which further exacerbated kidney inflammation, causing relentless interstitial fibrosis. NF-κB, which functions as a transcription factor for many miRNAs, has an important role in the regulation of countless cellular functions, including the cell cycle and apoptosis, among others^34^. in vitro inhibition of NF-κB significantly suppressed miR-382, indicating that NF-κB possibly targets miR-382. However, more in vivo intervention of NF-κB as well as RNA chip assays have to be conducted in the future. Moreover, whether miR-382 regulates the activation of inflammatory cells, such as macrophages, may demand further studies.

**Fig. 7:**
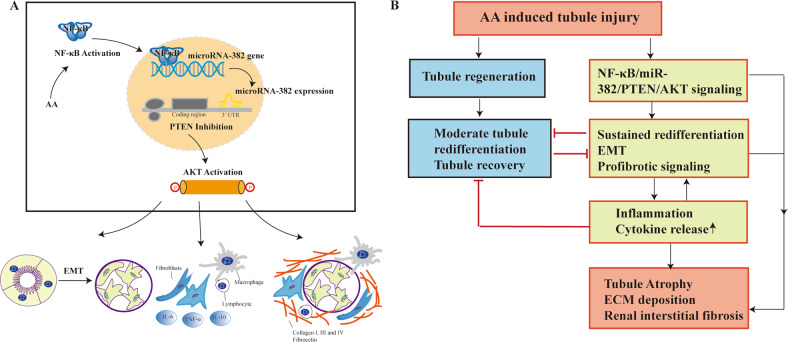
Possible working model of NF-κB/miR-382/PTEN/AKT signaling pathway in the progression of AA-induced AKI-to-CKD. **a** AA stimulation activated NF-κB, which acts as a transcriptional factor of miR-382 and promoted miR-382 expression. MiR-382 negatively regulated PTEN by combining with 3′UTR of *pten*. Inhibition of PTEN-activated AKT signaling pathway and then played roles in the progression of EMT, release of inflammatory cytokines and deposition of ECM. **b** Proposed the possible mechanism of AKI-to-CKD. AA treatment induced tubule injury, which would recovery after moderate tubule redifferentiation or would regulate NF-κB/miR-382/PTEN/AKT signaling axis resulting in sustained redifferentiation, EMT and expression of profibrotic signaling. In the process, inflammation response also promoted EMT process and prevented tubule recovery and finally led to tubule atrophy, deposition of ECM and renal interstitial fibrosis.

In conclusion, our study proposes a novel mechanism wherein overexpression of miR-382, which occurs subsequent to proinflammatory cytokine NF-κB activation, enhances AKI-to-CKD transition in mouse AA-induced kidney injury models (Fig. 7a, b).

## Materials and methods

### Mice

Male C57BL/6J mice (8- to 10-week-old; 20–25 g) were obtained commercially (SLAC LABORATORY ANIMAL CO. LTD, Shanghai, China). miR-382^−/−^ mice were bred in the BIORAY Lab, from C57BL/6 J mice back-crossed to miR-382^−/−^ mice, and used for experiments. All animal experiments were approved by the Institutional Animal Care and Use Committee of Fudan University and were performed in accordance with the National Institutes of Health Guide for the Care and Use of Laboratory Animals.

Aristolochic acid-induced AKI-to-CKD transition was conducted using an intraperitoneally injected single dose of Aristolochic acid I sodium salt (A9451, Sigma). All the mouse were randomly assigned to experimental groups. Samples were performed in biological replicates. The sample size was estimated based on the need for statistical power. We divided mouse into moderate AAN group (10 mg/kg) and severe AAN group (20 mg/kg) and mouse were separately killed at 1, 3, 7, 14, and 28 days (*n* = 6 mouse per group). Control mice were treated with the same dosage of saline via intraperitoneal injection. Locked nucleic acid- (LNA-) modified anti-miR-382 oligonucleotides or anti-scramble (Exiqon, Shanghai, China) at a dose of 10 mg/kg/7 d diluted in saline (5 mg/ml) was intraperitoneally administered to inhibit miR-382 expression in mice. The first injection was performed within 30 min prior to AA-I injection and the treatment was repeated every 7 d. All the experiments were replicated at least twice.

### Cell culture and siRNA transfection

Mouse renal tubular epithelial cells (mTECs) were purchased from the American Type Culture Collection (ATCC) and recently authenticated and tested for mycoplasma contamination. Cells were cultured in medium as indicated in Supplementary Table 2 and in a humidified chamber with 5% CO2 at 37 °C. Cells were cultured in 6-wells plates and randomly assigned to experimental groups. Cells were treated with AA (1, 2, 5, 10, or 20 μg/ml) for 48 h for concentration course study and with AA (6, 12, 24, 48, or 72 h) for 10 μg/ml for time course study. Transgene expression was induced with 100 nM anti-miR-382 (also referred to as anti-scramble) or 100 nM miR-382 mimic (also referred to as negative control) (Exiqon, Shanghai, China). Inhibition of NF-κB was performed by transfecting NF-κB siRNA or negative control (100 nM, Ribo Life Science, Shanghai, China). All the experiments were replicated at least twice.

### Human kidney tissue samples

We studied 12 patients with IgA nephropathy (IgAN) between 2017 and 2018 and performed renal biopsy in Zhongshan Hospital, Fudan University in order to evaluate tubulointerstitial fibrosis (TIF) in these patients using IgAN. Patients were divided into IgAN with TIF and IgAN without TIF groups (*n* = 6 per group) according to evaluation of a pathologist. The investigator was blind to the group allocation when immunohistochemical staining of PTEN and RT-qPCR of miR-382 were performed on human renal specimens. This study was approved by the Clinical Research Ethical Committee of the Zhongshan Hospital, Fudan University. Written informed consent was obtained from all patients.

### Serum creatinine

Serum creatinine levels of mice were determined using a QuantiChrom^TM^ Creatinine Assay Kit (BioAssay Systems, Hayward, CA, USA) following the manufacturer’s instructions.

### Immunohistochemical staining

Renal tissues were fixed with 10% formalin, embedded in paraffin wax and sliced into 4-μm-thick sections for hematoxylin–eosin (H&E) staining, Masson staining or immune-histochemical staining. Immunohistochemical staining was performed as described previously^13^. The primary and secondary antibodies were showed in Supplementary Table 2. Sections were evaluated via microscopy (×200 magnification, Leica DM 6000B; Leica Microsystems, Wetzeler, Germany).

### Immunofluorescence

After treatments, frozen kidney sections or mTECs, which crawled on the slide were fixed in 4% paraformaldehyde for 15 min. Cells were permeabilized by 0.5% Triton X-100 in PBS for 10 min. After blocking with 5% BSA in PBS, sections or cells were incubated with antibodies followed by secondary antibodies. Antibodies were presented in Supplementary Table 2. Images were acquired using Olympus FV1000 confocal microscope.

### Dual-luciferase assay

The targetScan bioinformatics website (http://www.targetscan.org/vert_71/) was utilized to predict targets of miR-382 and the possible sequence of the target binding sites with miR-382. Next, pMIR-PTEN-3′UTR-wt and pMIR-PTEN-3′UTR-mut were cloned into the pMiR dual-luciferase reporter plasmid vector. The recombinant pMiR dual-luciferase reporter plasmid was co-transfected with miR-382 mimics or negative control into 293T cells via lipo3000. Dual-luciferase activity was measured using the Dual-Glo Luciferase Assay System.

### Fluorescent miRNA in situ hybridization

Mmu-miR-382-5p in situ hybridization was performed using a customized kit according to the manufacturer’s instructions. Briefly, frozen sections were incubated with 4% PFA (containing 0.1% DEPC) for 30 min, proteinase (1 ml 3% citric acid + 2 drops concentrated proteinase) for 2 min at 37 °C, 1% PFA (containing 0.1% DEPC) for 10 min, 20 µl pre-hybridization solution for 2–4 h at 38–42 °C, hybridization solution overnight at 38–42 °C, and then washed SSC for 15 min at 37 °C, incubated with blocking buffer for 30 min at 37 °C, Digoxigenin-labeled LNA-modified probe corresponding to mature miR-382 for 1 h at 37 °C and Anti-Digoxigenin-Cy3 antibody for 1 h at 37 °C. Images were acquired using Olympus FV1000 confocal microscope.

### Western blotting

Western blot was performed as previously described^14^. The primary antibodies were incubated followed by HRP-conjugated secondary antibodies, which was showed in Supplementary Table 2. Protein levels were quantified using the Image Lab software, version 3.0 (Bio-Rad, USA).

### RNA isolation and real-time RT-PCR

Total RNA from mTECs, kidney tissues and biopsy specimens were extracted using Trizol, and reverse transcribed into cDNA using PrimeScript^TM^ RT reagent kit. The *18S rRNA* gene was used to normalize gene expression. Expression of miR-382 was detected using Taqman probes, using U6 to normalize miR-382 expression. Primer sequences used for PCR are shown Supplementary Table 1.

### Statistical analysis

All in vivo and in vitro experiments were performed in biological replicates. Data were analyzed using GraphPad Prism Software, and expressed as the mean ± standard error of the mean. Two-tailed, unpaired Student’s tests were performed to detect differences between two groups. The variance between the groups that are being statistically compared is similar. Statistical significance was set at *P* < 0.05.

## Supplementary information

Supplement Figure 1

Supplementary Information

Supplementary Table 1

Supplementary Table 2
