# Supplementary figures and images for: Upregulation of miR-382 contributes to renal fibrosis secondary to aristolochic acid-induced kidney injury via PTEN signaling pathway
Source: Cell Death Dis. 2020 Aug 14;11(8):620. doi: 10.1038/s41419-020-02876-1 (PMC7429500; doi:10.1038/s41419-020-02876-1)

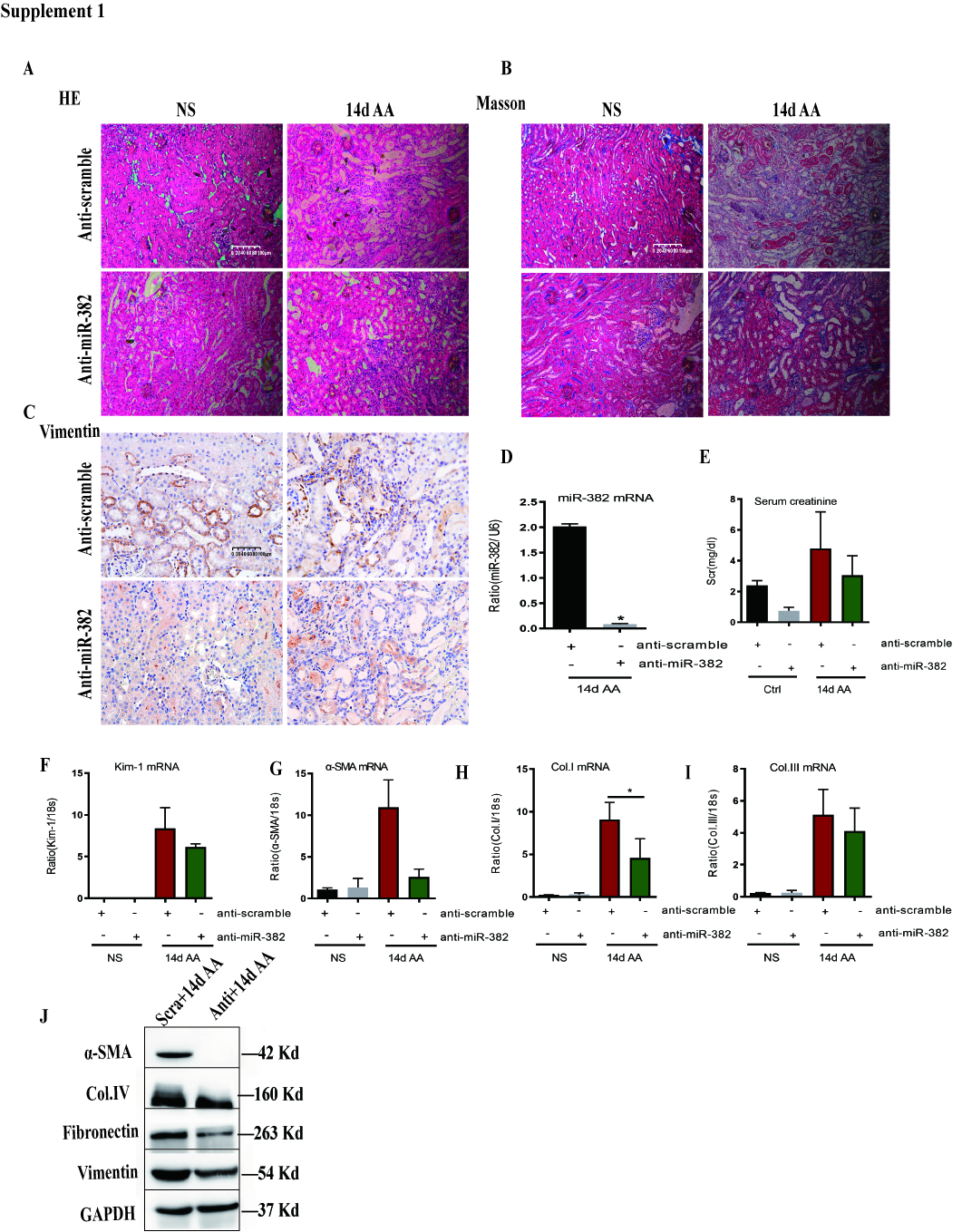

Supplement: Supplementary file 1 — Supplement Figure 1 [file 41419_2020_2876_MOESM1_ESM.tif]
