# Supplementary material for: Upregulation of miR-382 contributes to renal fibrosis secondary to aristolochic acid-induced kidney injury via PTEN signaling pathway
Source: Cell Death Dis. 2020 Aug 14;11(8):620. doi: 10.1038/s41419-020-02876-1 (PMC7429500; doi:10.1038/s41419-020-02876-1)
Supplement: Supplementary file 2 — Supplementary Information [file 41419_2020_2876_MOESM2_ESM.docx]

**Supplementary Information**

**Fig. S1: Inhibition of miR-382 contributes to reverse renal fibrosis in mice.**

**a-c** Representative images of kidney sections from anti-miR-382 or anti-scramble mice 14 d after the administering of 20 mg/kg AA (SAAN group) with H&E and Masson staining, as well as immunostaining for Vimentin. Scale bars, 100 μm. **d** Quantification of relative mRNA levels of miR-382 in mice kidney from anti-miR-382 or anti-scramble mice 14 days after the administering of 20 mg/kg AA (SAA group); n=6 per group; ^*^*P*< 0.05; ^**^*P* < 0.01; ANOVA. **e** Serum creatinine (Scr) levels between anti-miR-382 group, anti-scramble group, anti-miR-382+ 14 d SAAN group and anti-scramble+ 14 d SAAN group. **f-i** Administration of anti-miR-382 Oligo partially reversed kidney fibrosis. Relative mRNA expression of Kim-1, α-SMA, Collagen I and Collagen III in mice kidney tissues were examined between anti-miR-382 and anti-scramble group 14 d following the administering of 20 mg/kg AA. n=6 pre group; ^*^*P*< 0.05; ^**^*P* < 0.01. **j** Representative immunoblot analysis of α-SMA , Collagen IV, Fibroectin and Vimentin in renal from anti-miR-382 or anti-scramble mice 14 d following severe AAN. GAPDH served as the standard. AA, Aristolochic acid; α-SMA, α-smooth muscle actin; Kim-1, kidney injury molecular-1; GAPDH, glyceraldehyde-3-phosphate dehydrogenase.
