# Supplementary material for: Upregulation of miR-382 contributes to renal fibrosis secondary to aristolochic acid-induced kidney injury via PTEN signaling pathway
Source: Cell Death Dis. 2020 Aug 14;11(8):620. doi: 10.1038/s41419-020-02876-1 (PMC7429500; doi:10.1038/s41419-020-02876-1)
Supplement: Supplementary file 3 — Supplementary Table 1 [file 41419_2020_2876_MOESM3_ESM.docx]

**Supplementary Table 1.** Primer sequences used for real-time PCR analysis

| Target | Gene symbol | Sequence |
| --- | --- | --- |
| Nuclear factor kappa-B | NF-κB | Sense: 5’- AGGCTTCTGGGCCTTATGTG-3’,  Antisense: 5’- TGCTTCTCTCGCCAGGAATAC-3’ |
| Alpha-smooth muscle actin | α-SMA | Sense: 5’- CTGACAGAGGCACCACTGAA -3’,  Antisense: 5’- CATCTCCAGAGTCCAGCACA -3’ |
| Collagen I | Col. I | Sense: 5’- GAGCGGAGAGTACTGGATCG -3’,  Antisense: 5’- GTTCGGGCTGATGTACCAGT -3’ |
| Collagen III | Col. III | Sense: 5’- AGCACCTGTTTCTCCCTT -3’,  Antisense: 5’- CTGGTATGAAAGGACACAGAG -3’ |
| Interleukin-6 | IL-6 | Sense: 5’-TAGTCCTTCCTACCCCAATTTCC -3’,  Antisense: 5’-TTGGTCCTTAGCCACTCCTTC -3’ |
| Interleukin-10 | IL-10 | Sense: 5’-GCTCTTACTGACTGGCATGAG -3’,  Antisense: 5’-CGCAGCTCTAGGAGCATGTG -3’ |
| Tumor necrosis factor-α | TNF-α | Sense: 5’- CATGAGCACAGAAAGCATGATCCG -3’,  Antisense: 5’- AAGCAGGAATGAGAAGAGGCTGAG -3’ |
| 18s rRNA | 18s | Sense: 5’- CGGCTACCACATCCAAGGAA -3’,  Antisense: 5’- CCTGTATTGTTATTTTTCGTCACTACCT -3’ |
