# Supplementary material for: Upregulation of miR-382 contributes to renal fibrosis secondary to aristolochic acid-induced kidney injury via PTEN signaling pathway
Source: Cell Death Dis. 2020 Aug 14;11(8):620. doi: 10.1038/s41419-020-02876-1 (PMC7429500; doi:10.1038/s41419-020-02876-1)
Supplement: Supplementary file 4 — Supplementary Table 2 [file 41419_2020_2876_MOESM4_ESM.doc]

**Supplementary Table 2**. Antibodies and other reagents

| **Antibodies and reagents** | **Manufacturers** |
| --- | --- |
| Antibodies for western blot  Rabbit anti-human/mouse anti-α-SMA (ab5694, 1:1000)  Rabbit anti-human/mouse anti-Vimentin (ab137321, 1:1000)  Rabbit anti-human/mouse anti-PTEN (ab170941, 1:1000)  Rabbit anti-human/mouse anti-E-cadherin (#3195, 1:1000)  Rabbit anti-human/mouse anti-Collagen IV (50273S, 1:1000)  Rabbit anti-human/mouse anti-Fibronectin (F3648, 1:1000)  Rabbit anti-human/mouse anti-AKT (#4691, 1:1000)  Rabbit anti-human/mouse anti- p-AKT ser473 (#4060, 1:1000)  Rabbit anti-human/mouse anti-N-cadherin (13116S, 1:1000)  Rabbit anti-human/mouse anti- p-AKT thr308 (13038S, 1:1000)  Rabbit anti-human/mouse anti-GAPDH (ab181602, 1:2000)  HRP-conjugated goat anti-rabbit IgG Abs (#ZB-2301, 1:10000,) | Abcam, USA  Abcam, USA  Abcam, USA  Cell Signaling Technology, USA  Cell Signaling Technology, USA  Sigma-Aldrich, USA  Cell Signaling Technology, USA  Cell Signaling Technology, USA  Cell Signaling Technology, USA  Cell Signaling Technology, USA  Abcam, USA  Zhong Shan Gold Bridge Biotechnology, China |
| Antibodies for Immunohistochemistry  Rabbit anti-human/mouse anti-α-SMA (ab5694, 1:150)  Rabbit anti-human/mouse anti-Vimentin (ab137321, 1:150)  Rabbit anti-human/mouse anti-PTEN (ab170941, 1:50)  HRP-conjugated goat anti-rabbit IgG Abs (#ZB-2301, 1:10000,) | Abcam, USA  Abcam, USA  Abcam, USA  Zhong Shan Gold Bridge Biotechnology, China |
| Antibodies for Immunofluorescence  Rabbit anti-human/mouse anti-α-SMA (ab5694, 1:150)  Rabbit anti-human/mouse anti-NF-κB p-p65 (S536, 1:1000)  Rabbit anti-human/mouse anti-ZO-1 (ab96587, 1:200)  Rabbit anti-human/mouse anti-E-cadherin (#3195, 1:500)  Rabbit anti-human/mouse anti-Vimentin (ab137321, 1:150)  Donkey anti-Rabbit IgG-Alexa Fluor 488 (abs20020, 1:200)  Hoechst 33258 (1985358, 1:10000)  In situ hybridization probe  Digoxigenin-labeled LNA-modified probe corresponding to mature miR-382 (sequence: 5’-CGAAT CCACC ACGAA CAACT TC-3’)  Other reagents | Abcam, USA  Cell Signaling Technology, USA  Abcam, USA  Cell Signaling Technology, USA  Abcam, USA  Absin Bioscience Inc, Shanghai, China  Life technology, Shanghai, China |
| Aristolochic acid I sodium salt (A9451, Sigma)  lipo3000  Dual-Glo Luciferase Assay System  TRIzol  PrimeScript^TM^ reverse transcription Master Mix  TB® Premix Ex Taq^TM^  7500 real-time PCR System  Taqman probes  In situ hybridization kit (mmu-miR-382-5p)  High glucose DMEM  Fetal Bovine Serum  Penicillin-Streptomycin | Sigma-Aldrich, USA  Invitrogen, Shanghai, Chin  Promega, cat.no.E2920, E2940andE2980 (MDC)  Sigma-Aldrich, USA  Takara, Shanghai, China  Takara, Shanghai, China  Thermo Fisher Scientific, Pittsburgh, PA, USA  Applied Biosystems, Shanghai, China  Boster Biological technology, Shanghai, China  Gibco, Shanghai, China  Gibco, Shanghai, China  Gibco, Shanghai, China |
|  |  |
